# Disease models and AAV therapies of *PKP2*-induced arrhythmogenic cardiomyopathy

**DOI:** 10.1016/j.omta.2026.201688

**Published:** 2026-02-09

**Authors:** Lotte Geerlings, Yago Rodriguez Carreras, Mathilde C.S.C. Vermeer

**Affiliations:** 1 Department of Cardiology, University of Groningen, University Medical Center Groningen, Hanzeplein 1, 9700 RB Groningen, the Netherlands

**Keywords:** arrhythmogenic cardiomyopathy, *PKP2*, hiPSC, animal models, AAV gene therapy

## Abstract

Arrhythmogenic cardiomyopathy (ACM) is most commonly associated with pathogenic variants in the desmosomal gene plakophilin-2 (*PKP2*). However, *PKP2*-induced ACM remains challenging to decipher due to the multifaceted roles of its protein product, plakophilin-2 (PP2). This review provides a comparative analysis of experimental systems used to model *PKP2*-induced ACM, including *in vivo*, *ex vivo*, and *in vitro* animal models, as well as human induced pluripotent stem cell (hiPSC)-derived mono- and multicellular models. These models have revealed key insights into the roles of desmosomal dysfunction, lipid accumulation, and electrical instability during disease progression. Notably, therapeutic strategies aimed at restoring *PKP2* expression through adeno-associated virus (AAV) gene therapy have demonstrated promising results, suggesting a potential therapeutic approach for ACM. Notwithstanding these advances, challenges persist, including discrepancies between models, limited cardiomyocyte-specific models, and the need for further elucidation of the molecular pathways. This review emphasizes the importance of refining models to better capture the molecular landscape of *PKP2*-induced ACM, to enable improved mechanistic insight and the development of targeted therapies.

## Graphical abstract

**Figure:**
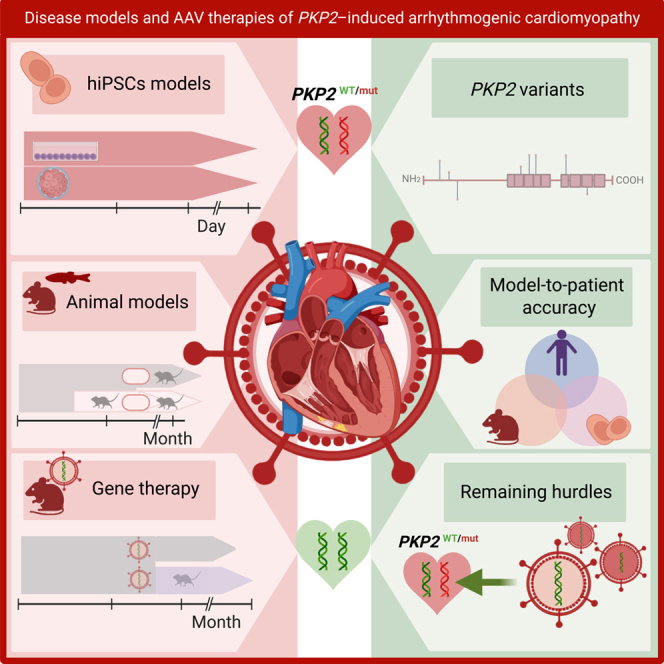

## Teaser

Vermeer and colleagues emphasize that, although murine models faithfully reproduce *PKP2*-induced ACM phenotypes *in vivo* and hiPSC systems capture genotypic complexity *in vitro*, neither currently integrates both features comprehensively. Bridging this gap is essential for enhancing mechanistic insight and advancing AAV-based gene therapies for patients.

## Introduction

**Figure 1:**
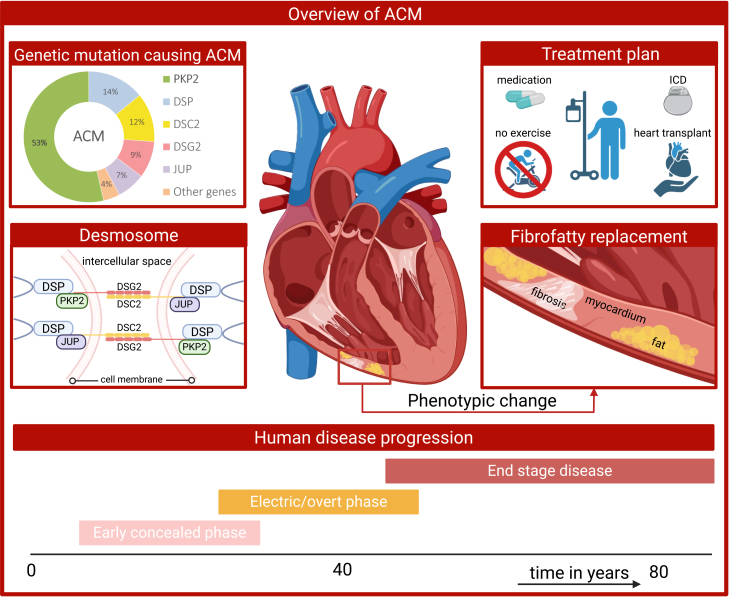
Overview of ACM Top-left: the contribution of desmosomal gene variants (in PKP2, plakophilin-2; DSP, desmoplakin; DSC2, desmocollin-2; DSG2, desmoglein-2; and JUP, plakoglobin) to the pathology of ACM. Mid-left: a zoom in of the desmosomal protein interactions. Top-right: the current treatment strategy for patients diagnosed with ACM (ICD, implantable cardioverter defibrillator,). Mid-right: schematic representation of the major hallmark of ACM, the fibro-fatty infiltration of the myocardium. Below: timeline of human disease progression in years, with the three main stages of disease depicted.

Arrhythmogenic cardiomyopathy (ACM) is a strictly inherited disease characterized by a predisposition to arrhythmias and fibro-fatty replacement of myocardial tissue.^1^^,^^2^ The fibro-fatty remodeling disrupts the electrical conduction of the heart and contributes to an even greater risk of ventricular arrhythmias as the disease progresses and the fibro-fatty areas increase.^3^^,^^4^^,^^5^ The prevalence of ACM is estimated to be 1:1,000–1:5,000, although undiagnosed cases and the risk of sudden cardiac death (SCD) complicate these figures.^3^^,^^6^^,^^7^^,^^8^ ACM is a leading cause of SCD in young adults, with one study assigning 20% of these events to ACM.^2^ The majority of ACM is caused by pathogenic variants in desmosomal genes plakophilin-2 (*PKP2*), desmoplakin (*DSP*), plakoglobin (*JUP*), desmocollin-2 (*DSC2*), and desmoglein-2 (*DSG2*) (Figure 1).^6^^,^^9^^,^^10^ Both the desmosome and intercalated disc are affected by these type of mutations. They play vital roles in maintaining the structural integrity and electrical synchrony of cardiomyocytes. This makes ACM a complex disease with both structural and electrical dimensions. These overlapping pathological mechanisms complicate efforts to model ACM effectively. The intricate network of interactions within the intercalated disc makes ACM particularly challenging to model and understand.^11^ As the understanding of ACM evolves, advances in *in vivo* and *in vitro* models are essential for elucidating the molecular and cellular mechanisms of ACM. Beyond mechanistic insights, such models provide a platform to test therapeutic strategies, including adeno-associated virus (AAV)-mediated gene therapies. While other reviews have highlighted insights into disease pathology,^11^^,^^12^ this review provides a comparative framework, focusing on the experimental setup of animal and human-derived models of *PKP2*-induced ACM in cardiac tissue. This review goes beyond prior summaries by comparing how animal and human-derived systems are engineered to model *PKP2*-induced ACM and by outlining how these platforms can accelerate the development of targeted gene therapies.

To inform this synthesis, the PubMed database was used to screen articles on the gene *PKP2*: first described in 1994, up to December 2024. Entries with the advanced search builder, using the terms “PKP2,” “PKP-2,” “Plakophilin-2,” “plakophilin 2,” and “Placophilin-2,” resulted in 796 articles. After title and abstract screening, pointing to the role of *PKP2* in the heart, 85 were selected for full-text review. Clinical studies were considered eligible if they investigated PP2 protein pathology (based on quantitative qPCR/RNA sequencing and/or quantitative western blotting) due to pathogenic variants in patient-derived material. Pre-clinical studies were considered eligible if they investigated *PKP2* (dys)function in the heart, or cell types native to and/or infiltrating the heart, of both animal and human origin. In total, 58 clinical and pre-clinical studies met the inclusion criteria and were incorporated in this review. Other relevant studies were included that contributed to the clinical background. All figures were created with BioRender.

## *PKP2-*induced ACM: Clinical manifestations and background

Autosomal dominantly inherited *PKP2* variants account for nearly half of all ACM cases and typically present in early adulthood with ventricular arrhythmias and an elevated risk of SCD.^8^^,^^13^^,^^14^^,^^15^^,^^16^ The early concealed phase is dominated by arrhythmias, while the overt/electric phase is marked by progressive fibro-fatty replacement, usually beginning in the right ventricle (RV). The end-stage phase shows severe biventricular fibro-fatty replacement and an increased risk of lethal arrhythmias. Patients typically have a less common risk to develop heart failure (HF, Figure 1).^17^ Compared with other desmosomal genes, dominant *PKP2* mutations associate with the “classic” right-dominant ACM phenotype. *DSP* mutations frequently cause proinflammatory early-onset biventricular or left-dominant failure, while *DSG2* mutations typically manifest with left-ventricular involvement and high risk of HF*. JUP* variants can also lead to dilated cardiomyopathy and HF, while *DSC2* mutations closely resemble the *PKP2-*associated phenotype.^18^^,^^19^^,^^20^^,^^21^^,^^22^^,^^23^^,^^24^

In contrast to the dominant *PKP2* variants, recessive mutations affecting both alleles cause earlier, more severe, and rapidly progressive disease.^25^^,^^26^ Most homozygous or compound heterozygous patients do not survive past infancy, presenting with *in utero* non-compaction and cardiac failure.^25^^,^^26^^,^^27^^,^^28^^,^^29^^,^^30^^,^^31^^,^^32^ Perinatal-onset biventricular dilated cardiomyopathy has been reported, often with fatal outcomes frequently occurring before fibro-fatty infiltration.^26^ These cases offer valuable insight into the effects of protein dosage on the severity of ACM. Although *PKP2* mutations have historically been considered structural, emerging evidence indicates that *PKP2*-related disease forms a mechanistic continuum extending into primary electrical disorders such as Brugada syndrome.^33^^,^^34^^,^^35^ Diagnosis of ACM relies on comprehensive clinical evaluation, with management strategies primarily targeting arrhythmic risk and prevention of SCD.^36^ Standard therapy focuses on arrhythmia control, lifestyle modification (avoidance of high-intensity exercise), and device-based prevention of SCD. HF-directed therapy is reserved for the minority of patients who develop late-stage ventricular dysfunction (Figure 1).^2^^,^^9^^,^^14^^,^^19^^,^^37^

Plakophilin-2 (furthermore referred to as PP2) is one of five desmosomal proteins that anchor adjacent cardiomyocytes and maintain myocardial integrity. Along with plakoglobin and desmoplakin, PP2 links the inner and outer dense plaques to transmembrane desmoglein-2 and desmocollin-2 (Figure 1). Desmosomes, core components of the intercalated disc, are in close proximity to fascia adherens, gap junctions, and ion channels, distributing mechanical forces across cardiomyocytes to prevent tissue rupture.^38^^,^^39^ Beyond structural support, desmosomes coordinate electrical propagation via NaV1.5 channels and Cx43 scaffolds, with PP2 directly interacting with these proteins. This dual structural and electrical role underlies how *PKP2* mutations compromise myocardial integrity and electrical stability in ACM.^11^

## Genetic and molecular basis of *PKP2*-induced ACM

**Figure 2:**
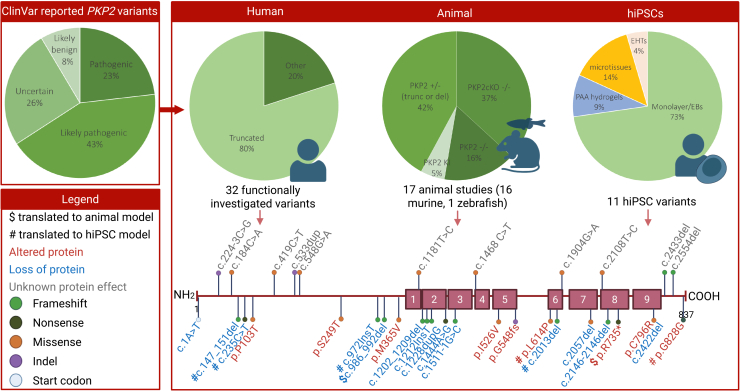
Reported *PKP2* variants in patients Top-left: pie-chart representation of the total reported amount of *PKP2* variants in the ClinVar database. Top-right: of these, 32 variants were functionally investigated in human biopsy samples, using western blotting and/or PCR techniques. Moreover, 17 animal models (16 murine and 1 zebrafish) and 11 hiPSC models were deployed. Bottom-right: all included variants were positioned in a lollipop representation of the *PP2* protein (9 armadillo repeats). Some of the variants overlap across these model systems, as indicated in the legend (bottom-left).

The human *PKP2* gene comprises 15 exons and produces two alternatively spliced isoforms: PP2a (92 kDa) and PP2b (97 kDa). The two isoforms differ in their splicing of exon 6, which encodes the final segment of the third armadillo repeat, which is unique to PP2b. In human heart muscle, only PP2a is expressed, which is furthermore referred to as PP2.^40^ The PP2 protein contains an HR2 domain and a conserved amino acid sequence in the N-terminal head domain, followed by nine armadillo repeats and a C-terminus.^41^ PP2 has been demonstrated to interact directly with desmosomal and intercalated disc proteins, such as desmoplakin and plakoglobin, with its N-terminal domain playing a critical role in these interactions.^42^ Pathogenic variants spanning the entire gene, including truncating and missense variants, have the capacity to significantly alter or disrupt the function of desmosomes. However, clinical descriptions indicate that 70%–90% of these variations are heterozygous truncating variants that lead to haploinsufficiency.^37^^,^^43^^,^^44^^,^^45^ These mutations induce a frameshift or splicing defect that leads to a premature termination codon. The latter is recognized by nonsense-mediated mRNA decay (NMD) pathways, which ultimately lead to a decrease in the total protein dose of functional PP2. Conversely, a much smaller proportion of ACM is induced by truncating variants that do not lead to NMD, missense variants, or other in-frame insertions or deletions. These result in functionally altered or impaired PP2 proteins and lead to varying degrees of clinical severity, depending on the alteration. The functional and clinical ramifications of these types of variants are by this definition harder to predict (Figure 2).

The dual structural and electrical roles of PP2 highlight the complexity of *PKP2*-induced ACM, posing challenges for disease modeling, which must capture defects in both myocardial integrity and electrical conduction. To explore these functional consequences, a range of experimental systems has been developed, including *in vivo* and *ex vivo* animal models, as well as *in vitro* models. The latter encompass animal-derived platforms, such as immortalized murine HL-1 cardiomyocytes and neonatal rat ventricular myocytes (NRVMs), and human induced pluripotent stem cell (hiPSC)-derived mono- and multicellular systems. Together, these approaches offer a complementary framework for studying *PKP2*-induced ACM.

## Animal models of *Pkp2-*induced ACM

**Figure 3:**
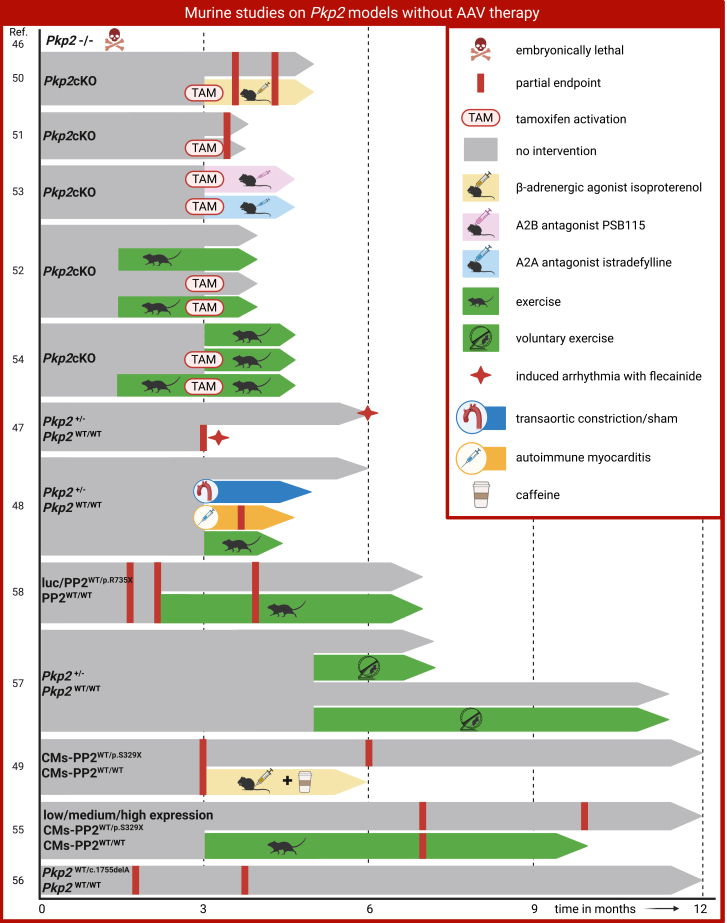
Murine studies on *Pkp2* models without AAV therapy Schematic representation of the mouse model designs used to model ACM development systemically. All interventions are depicted in the figure legend on the right. *x* axis is time in months; numbers on the *y* axis represent the study number references.^46^^,^^47^^,^^48^^,^^49^^,^^50^^,^^51^^,^^52^^,^^53^^,^^54^^,^^55^^,^^56^^,^^57^

A number of murine models of *PKP2-*induced ACM (gene *Pkp2*), as well as one zebrafish model (gene *pkp2*), have been developed and studied (see Figure 3; Table 1 and Table S1). In zebrafish embryos, early research utilized morpholino-mediated *pkp2* knockdown at varying doses, thereby generating a gradient of phenotypes from mild cardiac edema to severe ventricular malformations.^59^ In mice, the earliest whole-body knockout (*Pkp2*^−/−^, 0% wild-type PP2)^46^ proved embryonically lethal due to cardiac wall rupture.

**Table 1:** Animal models of PKP2-induced ACM and reported phenotypic characteristics

				Phenotypic characteristics							
Genetic manipulation	Animal model	Protein	Phenotype	RV dominant	Induced arrhythmia	Fibrosis accumulation	Altered desmosome expression	Altered Cx43 localization	Altered calcium handling	Altered sodium handling	Reference
*Pkp2 HOM*^−/−^	mouse	whole body: 0% WT PP2	embryonically lethal	–	–	–	–	–	–	–	Grossmann et al.^46^
*Pkp2 HET*^+/−^	mouse	50% WT PP2	–	+	+	–	–	–	+	+	Cerrone et al.^47^; van Opbergen et al.^48^
AAV-PP2^WT/p.R735X^	mouse	–	–	–	–	–	–	+	–	–	Cruz et al.^58^
*pkp2* gradual knockdown	zebrafish	gradual decrease PP2	increased severity due to knockdown	–	–	–	+	–	–	+	Moriarty et al.^59^
*Pkp2*^WT/c.1086InsT^ CMs-PP2^p.S329∗^	mouse	whole body: 50% WT PP2	Cardiomyopathy	+	+	–	+	–	+	–	Camors et al.^49^
CMs-*Pkp2*^*tamoxifen-KD*^ (*Pkp2*cKO)	mouse	30% WT PP2	SCD after dpi, mimics stages of disease	+	+	+	+	+	+	+	Cerrone et al.^50^; Kim et al.^51^; Cerrone et al.^52^; Cerrone et al.^53^; van Opbergen et al.^54^^,^^60^
*Pkp2*^WT/c.1086InsT^ CMs-PP2^p.S329∗^ (low, medium, high expression)	mouse	100% murine WT PP2 + overexpression human WT/human mutant PP2	mild changes in RV function and structure; decreased RVOT	+	+	–	+	+	–	+	Moncayo-Arlandi et al.^55^
*Pkp2*^*WT/c.1755delA*^	mouse	whole body; 50% WT PP2	impaired ventricular relaxation; normal EF	–	–	+	+	–	–	–	Tsui et al.^56^^,^^61^
CMs-*Pkp2* ^c.2146G>C/c.2416G>C^	mouse	0% WT PP2, 30% mutant PP2	HOM, dies before 26 weeks of age	+	+	+	+	+	–	+	Bradford et al.^62^

To approach the dominant phenotype of patients, a heterozygous *Pkp2*^+/−^ was developed to model human *PKP2* haploinsufficiency. These animals demonstrate survival into adulthood displaying only mild or delayed phenotypes under baseline conditions.^47^^,^^48^^,^^57^ In a similar manner, knockin models carrying patient-specific *Pkp2* variants have been generated to reproduce a heterozygous genetic background. For instance, a whole-body knockin line created using CRISPR-Cas (*Pkp2*^WT/c.1755delA^, with 50% wild-type PP2) exhibited only mild structural changes with limited fibrosis and did not show overt HF or SCD within the first year of life.^56^^,^^61^ Another patient-specific variant has been the subject of study through AAV-mediated, cardiac-specific expression of the human genotype *PKP2*^WT/c.2203C>T^ (referred to as PP2^WT/p.R735X^, expressing the mutant protein), allowing evaluation of genetic effects under exercise conditions.^58^ Furthermore, transgenic mice expressing a truncated PP2 protein (mutation *Pkp2*^WT/c.1086InsT^ referred to as cardiomyocyte-specific [CMs] CMs-PP2^p.S329∗^) have been observed to develop progressive cardiomyopathy, ventricular arrhythmias, and structural remodeling consistent with ACM.^49^ In addition, graded transgene expression has been shown to modulate disease severity.^55^

In order to better model human disease progression, a tamoxifen-inducible and cardiomyocyte-specific *Pkp2* knockout mouse model (*Pkp2*cKO) has been developed. Following tamoxifen administration at 3 months of age, there was a significant decrease in murine PP2 levels, decreasing up toto 30% of original levels. This resulted in a high risk of SCD in the weeks following induction. Furthermore, *Pkp2*cKO mice began to manifest progressive cardiac dysfunction and arrhythmias between 3 and 6 months of age.^50^^,^^51^^,^^52^^,^^53^^,^^54^^,^^60^^,^^63^^,^^64^ Most recently, a cardiomyocyte-specific knockin model carrying the human splice-site mutation IVS10-1G>C (CMs-*Pkp2*^WT/c.2146G>C^ and CMs-*Pkp2*^c.2146G>C/c.2146G>C^) was generated. Homozygous animals, lacking all wild-type PP2, exhibited hallmark features of ACM, including ventricular dilation, conduction delays, fibrosis, and arrhythmias, and generally died before 26 weeks of age. Although the researchers reported limited adipocyte deposition in these mice, it is important to note that fibro-fatty replacement, as observed in human ACM, remains minimal and regionally restricted in murine hearts. Heterozygous animals were unfortunately not extensively studied.^62^ Together, these models provide a versatile toolkit for the systemic study of *Pkp2*-induced ACM, in addition to the evaluation of therapeutic interventions.

## Dosage-dependent phenotypes in *Pkp2 in vivo* models

The embryonic lethality of the whole-body knockout/knockdown of *Pkp2* in both murine and zebrafish^46^^,^^59^ is consistent with data on biallelic inheritance in patients,^32^ highlighting the essential role of PP2 in early cardiac development. Zebrafish with gradual knockdown of *pkp2* demonstrated a spectrum of developmental failure^59^ (see Table 1). By contrast, heterozygous mice or knockin models with ∼50% *Pkp2* expression (haploinsufficiency) exhibit only subtle or delayed phenotypes: mild contractile dysfunction, limited fibrosis, or altered intercalated disc configurations, without overt HF or SCD.^48^^,^^65^^,^^66^ This is contrasting to patients with *PKP2* haploinsufficiency, who are at high risk of SCD during adolescence or young adulthood. Such discrepancies may reflect species-to-species differences in extracellular matrix turnover, calcium handling, or strain background.^67^^,^^68^ Notably, several haploinsufficient models showed reduced levels of calcium-handling proteins (ankyrin-B, calsequestrin, and SERCA2a) and degradation of intercalated disc proteins, aligning with patient findings but failing to reproduce the full clinical spectrum of disease. Owing to their mild phenotype, these studies typically required extended follow-up^47^^,^^49^^,^^56^^,^^61^ (see Figure 3; Table 1). Clinical hallmarks such as fibro-fatty replacement usually manifest in later stages of human disease, suggesting that the findings in haploinsufficient mice may resemble the more early stages of human ACM, but these models still lack the risk for SCD.^44^ Furthermore, only one of the aforementioned models exhibited electrical alterations when subjected to isoproterenol or exercise.^49^

Stronger concordance between animal models and patients emerges when wild-type PP2 protein levels fall below 50%. More severe models, including the *Pkp2*cKO (∼30% protein expression) and CMs-*Pkp2*^c.2146G>C/c.2146G>C^ mouse model, did develop fibrosis, RV dilation, contractile dysfunction, and arrhythmias. These features closely mirrored human disease progression, particularly under exercise and/or isoproterenol administration^52^^,^^54^^,^^62^ (see Table 1). Among these, the *Pkp2*cKO model recapitulated different disease stages with striking fidelity. At 14 days post induction (dpi), mice exhibit dilated RV with normal left ventricular ejection fraction (LVEF), and an increased risk in arrhythmias was observed. The initial phase following the activation of tamoxifen also coincided with a high risk of SCD. By 21 dpi, RV dilation progresses, LVEF modestly declined (±55%), and electrical alterations and premature ventricular contractions had emerged. By 42 dpi, both ventricles were dilated, the LVEF had dropped to ±20%, and a high mortality rate was observed, consistent with end-stage biventricular failure.

Importantly, *Pkp2*cKO represents a partial knockout, achieving roughly 70% depletion of murine PP2 (with residual expression of ∼30%), shortly after induction, likely reflecting incomplete Cre-mediated recombination across cardiac cell/tissue populations. Despite this limitation, the resulting PP2 dosage closely mirrors the functional consequences of *PKP2* haploinsufficiency in patients and more accurately reproduces disease severity than murine models retaining approximately 50% PP2 expression, which display substantially milder phenotypes.^10^^,^^51^^,^^52^^,^^53^^,^^54^

*In vivo* animal models have consistently demonstrated that desmosomal dysfunction leads to impaired mechanical coupling of cardiomyocytes, resulting in compromised cellular adhesion and structural integrity.^46^^,^^59^ This mechanical uncoupling initiates a cascade of pathological remodeling, including fibrosis and fatty infiltration, which further disrupt myocardial architecture and function.^48^^,^^65^^,^^66^ Additionally, inflammatory responses and altered electrical properties, such as changes in sodium channel function and gap junction remodeling, have been documented.^54^^,^^56^ Another study suggests proteasomal degradation pathways affecting intercalated disc proteins disproportionally as significant contributors to disease progression. Indeed, inhibitory treatments of the proteasome have increased many of the decreased intercalated disc proteins.^56^ Together, these mechanisms act in synergy to generate an arrhythmogenic substrate that predisposes to ventricular arrhythmias and HF, with calcium-handling defects emerging as an important downstream or parallel contributor, discussed in detail in the following section.

## *Ex vivo* mechanistic insight from animal models

*Ex vivo* and isolated cardiomyocyte studies have been instrumental in dissecting cardiomyocyte-autonomous mechanisms downstream of PP2 deficiency.^50^ Transcriptomic and phospho-proteomic analyses revealed coordinated reduced expression of calcium-related genes, including *Ank2*, *Ryr2*, and *Cacna1c.* This was accompanied by prolonged calcium transients, action potential duration, and the emergence of early- and delayed-after depolarizations.^50^

In cardiomyocytes derived from *Pkp2*cKO mice, PP2 loss resulted in calcium accumulation in the sarcoplasmic reticulum, mitochondria, and cytoplasm, thereby disrupting calcium homeostasis. Phospho-proteomic profiling further implicated impaired phosphorylation signaling as a contributor to calcium-handling defects. This imbalance was linked to a reduction in *GJA1* expression, which impaired the stability of gap junctions, promoting increased membrane permeability through Cx43 hemichannels and exacerbating calcium overload. Importantly, these disturbances preceded overt structural remodeling and contributed to arrhythmia development during disease progression.^51^^,^^69^

In the longest non-intervention study on *Pkp2*cKO mice, mortality reached nearly 100% by 42 dpi.^52^^,^^54^ Although isolated *Pkp2*-deficient cardiomyocytes displayed enhanced sarcomeric shortening and elevated calcium transient amplitude *ex vivo*, these hypercontractile features did not translate *in vivo*, where progressive cardiomyocyte loss, myocardial wasting, and ventricular dysfunction dominated disease progression. Exercise and β-adrenergic stimulation further amplified calcium instability, with increased spontaneous calcium release events and sarcoplasmic reticulum loading, reinforcing *Ryr2*-mediated dysfunction as a central driver of arrhythmogenesis.^54^ Complementary studies in human cardiomyocytes confirmed that *PKP2* knockdown alters sodium current properties and slows action potential propagation,^74^ supporting a cardiomyocyte-autonomous electrical substrate that contributes to disease initiation and progression.

## Gene therapy in murine models

**Figure 4:**
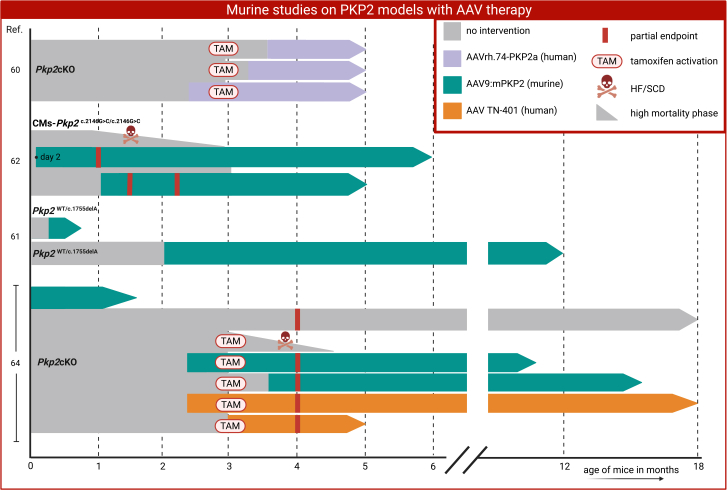
Murine studies on *Pkp2* models with AAV therapy Schematic representation of the mouse model designs that used AAV *PKP2* replacement therapy. All interventions are depicted in the figure legend on the right. AAV, adeno-associated virus; CM, cardiomyocyte; HF, heart failure; SCD, sudden cardiac death; WT, wild type. *x* axis is time in months; numbers on the *y* axis represent the study number references.^60^^,^^61^^,^^62^^,^^64^

Two studies pioneered in 2023 with AAV *Pkp2* gene therapy, which included the whole-body heterozygous *Pkp2*^WT/c.1755delA^ knockin and the homozygous CMs-*Pkp2*^c.2146G>C/c.2146G>C^ mouse model^56^^,^^61^^,^^62^ (Figure 4). In the heterozygous, phenotypically mild *Pkp2*^WT/c.1755delA^ model, presymptomatic human *PKP2* AAV administration resulted in restoration of PP2 levels, which prevented disease onset. A single dose administered at 2 months of age prevented the development of cardiac dysfunction up to 12 months. However, the absence of hallmark ACM features such as SCD or progressive LVEF decline limits the model’s ability to assess therapeutic efficacy across the full disease spectrum.^61^

In contrast, the non-intervention homozygous CMs-*Pkp2*^c.2146G>C/c.2146G>C^ mouse model (lacking wild-type PP2) exhibited rapid disease progression with a median lifespan of 11 week. In this setting, early presymptomatic AAV-*PKP2* treatment (day 2) secured 100% survival up to 6 months. Treatment initiated at 1 month of age partially preserved LVEF but was less effective than early intervention, highlighting the importance of therapeutic timing.^62^

Two subsequent studies employed the *Pkp2*cKO mouse model, using 3 types of AAVs.^60^^,^^64^ One study employed a human *PKP2* vector (AAVrh.74 PKP2a) administered at multiple time points before and after tamoxifen induction. Treatment restored *PKP2* mRNA levels, prevented HF, and ensured survival up to 5 months post treatment. Notably, administration at 14 dpi, corresponding to an early disease stage, provided a more physiologically relevant intervention window than phenotypically mild knockin models, although efficacy in advanced disease remained untested.^60^ A second study compared murine (AAV9:m*Pkp2*) and human (TN 401) vectors. While untreated mice exhibited complete mortality by 42 dpi, gene therapy delivered before tamoxifen activation markedly improved survival, reduced arrhythmic burden, and preserved cardiac structure and function. Importantly, a single post-induction treatment at 3 weeks halted disease progression and improved LVEF, aligning more closely with an earlier stage human disease scenario^64^ (Figure 4).

Collectively, these studies demonstrate the potential of AAV gene therapy in mitigating ACM progression, but they also emphasize key challenges. Clinically relevant treatment occurs after disease onset, whereas most murine interventions remain presymptomatic.^60^^,^^62^ Moreover, the specificity of many AAV therapies to cardiomyocytes, applied mostly to cardiomyocyte-specific *Pkp2* mouse models, raises concerns about overlooking the role of non-cardiomyocytes in the development of ACM. This may constrain the potential for broader therapeutic translation to human clinical trials. Indeed, the only whole-body model that was used was phenotypically mild, complicating the translation to patients.

Finally, while systemic *in vivo* models capture disease progression and therapeutic response, they are poorly suited for resolving the earliest cardiomyocyte-autonomous consequences of *Pkp2* deficiency. To address these primary mechanisms, early *in vitro* studies turned to animal-derived cardiomyocyte systems, including NRVMs and HL1 cells.

## *In vitro* mechanistic insights from animal-derived cell lines

Prior to the widespread use of hiPSC-derived cardiomyocytes, important mechanistic insights into *Pkp2* function were obtained from HL-1 atrial cells and NRVMs. Although these systems do not recapitulate human ventricular maturation or disease progression, they were instrumental in defining the molecular pathways linking PP2 deficiency to impaired electrical coupling, cytoskeletal remodeling, and adhesion-related signaling.

In HL-1 cells, short hairpin RNA (shRNA)-mediated reduction of *Pkp2* demonstrated a selective decrease in sodium current due to impaired Nav1.5 localization at junctions.^34^ *Pkp2* knockdown also altered adhesion-related signaling by increasing *miR-200b* expression and suppressing *Itga1*, consistent with impaired mechanosensing.^70^ Broader work in HL-1 cells further described non-junctional pools of *Pkp2* and its presence in additional cell contact structures.^71^ More recently, automated single-cell manipulation approaches using HL-1 identified modifiers of gap junction function,^72^ and HL-1 fibroblast co-culture studies demonstrated a role for *Dlk1-Notch* signaling in fibro-fatty replacement.^73^

NRVM studies provided complementary mechanistic evidence. *Pkp2* silencing strategies reduced total Cx43 levels and redistribution to intracellular compartments,^69^ while also decreasing sodium current amplitudes and conduction velocities.^74^ Expression of *Pkp2* mutants on the other hand disrupted junctional protein localization and cytoskeletal structure.^75^ Additional work confirmed that PP2, ankyrin G, and Cx43 coexist in the same macromolecular complex at the intercalated disc.^76^ Experimental and computational analyses further showed that changes in sodium current kinetics were the dominant trigger for rotor initiation and conduction instability in *Pkp2*-deficient monolayers.^77^ Together, results from HL-1 and NRVM models established that *Pkp2* regulates sodium channel localization and kinetics, maintains gap junction stability, and contributes to cytoskeletal and adhesion-related signaling.

## hiPSC models of *PKP2*-induced ACM

**Figure 5:**
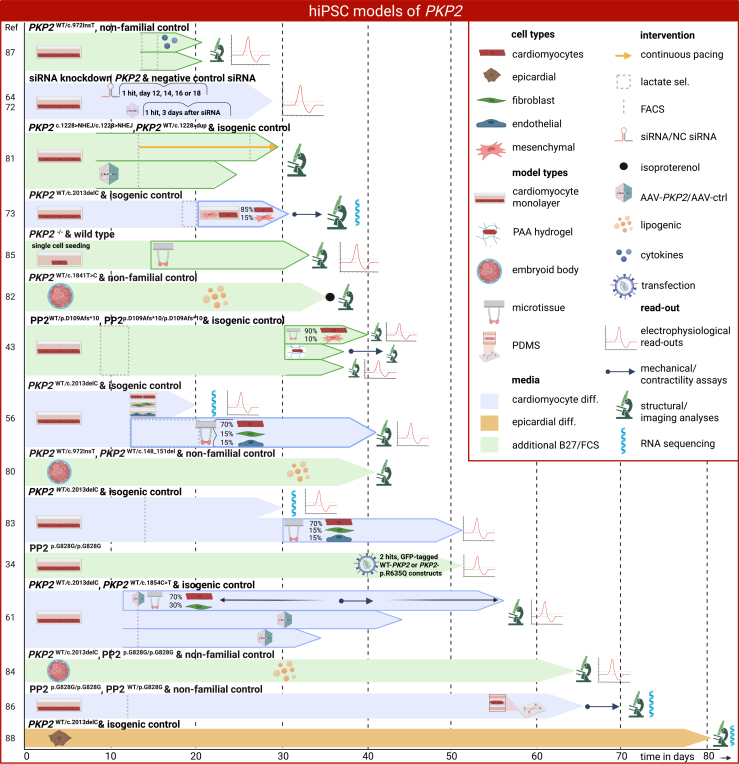
hiPSC models of *PKP2* Schematic representation of hiPSC model designs of *PKP2*-induced ACM, including the use of AAV *PKP2* replacement therapy. AAV, adeno-associated virus; NC, negative control; PAA, polyacrylic acid; siRNA, silencing RNA. *x* axis is time in days; numbers on the *y* axis represent the study number references.

hiPSC models offer several advantages for studying *PKP2*-induced ACM, as they can proliferate indefinitely and have diverse differentiation abilities. This allows for a multitude of experimental setups to study disease progression in long-term studies^78^^,^^79^ (schematic overview in Figure 5). For *PKP2-*induced ACM, the following variants causing haploinsufficiency—*PKP2*^WT/c.148_151del^, *PKP2*^WT/c.972insT^, *PKP2*^WT/c.1228dup^, *PKP2*^WT/c.1841T>C^, *PKP2*^WT/c.1854C>T^, *PKP2*^WT/c.2013del^ (all with 50% wild-type PP2)—have been studied from patient-derived hiPSCs.^61^^,^^80^^,^^81^^,^^82^^,^^83^^,^^84^ Meanwhile, variants *PKP2*^c.1228>NHEJ/c.1228>NHEJ^ (edited with non-homologous end joining from *PKP2*^WT/c.1228dup^ now producing 0% wild-type PP2) and a non-human variant on either or both alleles (*PKP2*^WT/c.326del5bp^ and *PKP2*^c.326del5bp/c.326del5bp^ either producing 50% or 0% wild-type PP2) have been engineered in hiPSCs using CRISPR-Cas.^43^^,^^81^ One study generated *PKP2*^−/−^ hiPSCs from wild-type C hiPSCs where out-of-frame indels resulted in premature stop codons, resulting in a full knockout lacking any PP2 production.^85^ Another model used silencing RNA (siRNA) knockdown of *PKP2* in control hiPSC-cardiomyocytes, which has been utilized in two studies.^64^^,^^72^ While all of the aforementioned variants significantly lower the dose of wild-type PP2, only one variant has been investigated that produces an alternate PP2 protein. Variant *PKP2*^c.2484C>T/c.2484C>T^ causes cryptic splicing with a 7-nucleotide deletion in exon 12, leading to frameshift of the C-terminal amino acids. In total, 80% of PKP2 mRNA was cryptically spliced, which resulted in abundant expression of the C-terminal truncated protein (PP2^p.G828G-fs/p.G828G-fs^) in hiPSC-cardiomyocytes, while only 20% of mRNA was normally spliced causing only small amounts of wild-type PP2 protein^34^^,^^86^ (Table 2; Figure 5).

**Table 2:** hiPSC models of PKP2-induced ACM and reported phenotypic characteristics

			Phenotypic characteristics							
HGVS nomenclature (DNA)	Protein	Type of mutation	Cell death	Lipid accumulation	Altered cell metabolism	Altered desmosome expression	Altered Cx43 localization	Altered calcium handling	Altered sodium handling	Reference
*PKP2*^WT/c.148_151del^*PKP2*^WT/c.972insT^	40% WT	indel-nonsense	+	+	–	+	+	–	–	Caspi et al.^80^; Morrissette-McAlmon et al.^87^
PP2^WTc.326del5bp^	50% alternativep.D109A	frameshift (CRISPR engineered)	–	–	–	+	+	–	–	Zhang et al.^43^
PP2^c.326del5bp/c.326del5bp^	25% alternativep.D109A	frameshift (CRISPR engineered)	–	–	–	+	+	+	–	Zhang et al.^43^
*PKP2*^c.1841T>C^	50% WT	missense	–	+	–	+	–	–	–	Ma et al.^82^
*PKP2*^WT/c.1228dupG^	50% WT	frameshift	–	–	–	+	+	–	–	Inoue et al.^81^
*PKP2*^c.1228NJEH/c.1228NJEH^	0%	frameshift	+	+	–	+	+	–	–	Inoue et al.^81^
*PKP2*^WT/c.2013delC^	50% WT	indel-nonsense	+	+	–	+	+	+	+	Tsui et al.^56^; Kyriakopoulou et al.^61^; Maione et al.^73^; Giacomelli et al.^83^; Kim et al.^84^; Kohela et al.^88^
*PKP2*^WT/c.1854C>T^	50% WT	indel-nonsense	+	+	+	–	+	+	+	Tsui et al.; Kyriakopoulou et al.^61^
*PKP2*^c.2484C>T/c.2484C>T^	∼20% alternativep.G828G:GGC>GGT	frameshift	–	+	–	–	+	+	+	Cerrone et al.^34^
*PKP2*^−/−^	0% WT	indel-nonsense	–	–	–	+	+	+	+	Simmons et al.^85^

### Patient-specific undefined multicellular hiPSC-derived models

Several studies have leveraged hiPSC-derived models to investigate the impact of *PKP2* mutations, with foundational work in 2013 establishing embryoid body (EB) formation from multiple hiPSC lines.^80^^,^^82^^,^^84^ These EBs contain differentiated cardiomyocytes alongside other undefined cell types from ectodermal, endodermal, and mesodermal origins, all sharing the patient’s genetic background. The first EB study with either a cryptic splicing mutation (*PKP2*^c.2484C>T/c.2484C>T^, 20% wild-type PP2 with abnormal PP2) or a frameshift mutation (*PKP2*^WT/c.2013delC^, 50% wild-type PP2) revealed significant disruptions in nuclear translocation of plakoglobin and a reduction in β-catenin activity compared to control EBs. Under metabolic conditions mimicking more adult-like cardiomyocyte energetics (pathways related to PPAR-α were co-activated alongside an abnormal pathway related to the PPAR-γ), these EBs exhibited exaggerated lipogenesis, apoptosis, and calcium-handling deficits over 60 days, implicating altered lipid metabolism and cellular stress in the pathogenesis of ACM.^84^^,^^89^ However, the lack of appropriate maturation controls, such as adult cardiomyocyte fatty acid uptake assays, limits these interpretations. Complementing these findings, the second EB study on the *PKP2*^WT/c.1841T>C^ (50% wild-type PP2) mutation, reported similar plakoglobin delocalizations, β-catenin downregulation, and calcium-handling abnormalities. Transmission electron microscopy revealed lipid droplet accumulation exacerbated by adipogenic stimulation, further linking metabolic dysregulation to disease.^82^ The third EB study used two hiPSC-derived EB models with either *PKP2*^WT/c.972InsT/N^ (40% wild-type PP2) or *PKP2*^WT/c.148_151delACAG/N^ (40% wild-type PP2) variants, confirmed desmosomal abnormalities, reduced plakoglobin and lipid droplet accumulation, and elevated *PPAR-γ* expression. Notably, increased desmosomal gap size correlated with lipid accumulation, which was suppressible via canonical Wnt pathway activation, highlighting a potential therapeutic axis.^80^ Collectively, these studies identify desmosomal dysfunction, altered intracellular signaling, and metabolic stress as concurrent and central features of *PKP2*-mutant EBs. Notably, the reduction in plakoglobin consistently observed in hiPSC models has not been reported in murine systems (Tables 1 and 2), although alterations in other intercalated disc proteins, such as Cx43, are evident in patient tissue.^80^

Further mechanistic clarity is provided by evidence that *PKP2* loss affects electrical function before the occurrence of overt structural remodeling. Missense mutations have been demonstrated to directly impair Nav1.5 trafficking and reduce peak sodium current, defining an early electrical phenotype that arises independently of fibro-fatty changes.^34^^,^^74^ This underscores a pivotal link between desmosomal integrity and sodium channel localization, establishing a mechanistic linkage between the desmosomal abnormalities observed in EBs and the early arrhythmogenic risk manifesting clinically. Collectively, these findings emphasize that *PKP2* deficiency disrupts the structural, metabolic, and electrical domains in a concurrent manner and that elucidating their temporal sequence remains a pivotal endeavor in comprehending the molecular underpinnings of ACM.^34^

Despite these shared phenotypes between EBs and patients, the causal hierarchy remains unresolved, including whether metabolic rewiring and PPAR-γ activation drive secondary junctional instability or whether primary desmosomal insufficiency initiates downstream metabolic and transcriptional remodeling. While EB-based systems indicate that PP2 deficiency simultaneously affects structural, metabolic, and electrical domains, their cellular heterogeneity limits attribution of early phenotypes to specific cell types. Meanwhile, more directed hiPSC differentiation protocols quickly emerged, and because ACM was long regarded as a desmosome-driven disease, emphasis shifted toward cardiomyocyte purification methods.

### Patient-specific monocellular hiPSC-derived models

Building on these foundations, more recent studies have used directed cardiac differentiation protocols w/o lactate selection to generate purified cardiomyocyte populations, enabling refined mechanistic analyses. For example, hiPSC-cardiomyocytes with a nonsense frameshift mutation (*PKP2*^WT/c.1228dup^, 50% wild-type PP2, and a knockout derivative *PKP2*^c.1228>NHEJ/c.1228>NHEJ^, no PP2) exhibited cell-cell adhesion under contractile stress, accompanied by increased apoptosis and reduced troponin-T expression, underscoring impaired cardiomyocyte function and survival. *PKP2* insufficiency also reduced cardiomyocyte contractility, with decreased contraction velocities and deformation compared to control.^81^ Furthermore, lactate-purified monolayers harboring the *PKP2*^c.2484C>T*/*c.2484C>T^ mutation (referred to as PP2^p.G828G/p.G828G^) exhibited altered expression of ∼800 genes under uniaxial stretch, including those related to mechanic transduction, reinforcing the role of PP2 in cellular responses to mechanical stress.^86^ Additionally, *PKP2*^WT/c.972InsT^-mutated cardiomyocyte monolayers demonstrated altered NF-κB signaling upon paracrine stimulation, suggesting that cytokine responses and crosstalk with non-myocyte cells such as infiltrating adipocytes may modulate the arrhythmogenic phenotype.^87^ These data together underscore the importance of PP2 dosage on the phenotypic characteristics observed in human *in vitro* models and also show that secondary factors such as stretch or paracrine factors from other cell types may influence phenotypic outcomes.^86^^,^^87^

Mechanical environment further modulates *PKP2*-dependent dysfunction. Beyond the use of cardiomyocyte monolayers, engineered three-dimensional so-called “micro-heart muscle arrays” demonstrated that tissue geometry and *PKP2* expression co-regulate Nav1.5 function, indicating that structural constraints alone can unmask sodium current deficits in *PKP2*-mutant hiPSC-cardiomyocytes.^85^ Moreover, robotic single-cell manipulation has shown mechanically regulated gap-junction coupling in *PKP2*-deficient hiPSC-cardiomyocytes, linking interface topology to Cx43 trafficking and conduction vulnerability.^72^ These findings reinforce the concept that *PKP2* deficiency profoundly alters how cardiomyocytes integrate mechanical and electrical cues.

In this context, it is notable that early EB-based models captured adipogenic phenotypes, whereas lactate-selected hiPSC-cardiomyocyte monocultures did not. This contrast highlights a central trade-off in *in vitro* modeling of *PKP2*-induced ACM: undefined multicellular EBs can reproduce disease features that depend on non-cardiomyocyte populations but do not allow phenotypes to be attributed to specific cell types. However, the limitations inherent in these models preclude the ability to assign causality to specific cell types. In contrast, purified cardiomyocytes enable precise mechanistic research, yet may mask phenotypes that emerge only through multicellular interactions. Together, these limitations motivated the development of defined multicellular hiPSC models that bridge cardiomyocyte-intrinsic mechanisms with the broader cellular context of ACM.

### Defined multicellular hiPSC-derived models of mixed genetic background

Increasingly complex *in vitro* systems have begun to incorporate multiple cardiac cell types and engineered three-dimensional tissue architectures, enabling investigation of how PP2 deficiency interacts with tissue geometry, mechanical load, and electrical coupling. These defined multicellular models, while not patient specific across all cell types, provide an essential intermediate step by isolating how structural context and cell-cell interactions shape phenotype expression. One study compared both the CRISPR-Cas9-engineered biallelic PP2^p.D109Afs∗10/p.D109Afs∗10^ (20% wild-type PP2) and monoallelic PP2^WT/p.D109Afs∗10^ (50% wild-type PP2) mutation in hiPSC-derived lactate-selected cardiomyocyte monolayers, microtissues (90% hiPSC-derived cardiomyocytes and 10% human mesenchymal cells), and polyacrylamide hydrogels.^43^ Cardiac microtissues showed electrical instability and susceptibility to arrhythmias, as observed by a prolonged action potential. Interestingly, both cardiac microtissues and cardiomyocyte monolayers had decreased contractility in biallelic mutated tissues, while single-cell seeded cardiomyocyte counterparts showed increased contractility. The decrease in contractility in monolayers and microtissues could be attributed to impaired junction stability, which in turn affects the sarcomere stability and organization. These results illustrate how tissue architecture shapes phenotype and highlight the challenges of comparing findings across model systems (Figure 5).

### Patient-specific defined multicellular hiPSC-derived models

While mixed genetic multicellular systems clarify how *PKP2* deficiency perturbs the integration of mechanical and electrical cues at the tissue level, complete patient-derived models are required to determine how individual cardiac cell types actively contribute to disease initiation and progression. Multicellular hiPSC systems have been particularly informative in this regard. In microtissues incorporating patient-specific cardiomyocytes, fibroblasts, and endothelial cells, *PKP2*^WT/c.2013delC^ mutation (50% wild-type PP) fibroblasts alone were sufficient to impair pacing response and induce arrhythmia-like behavior, underscoring the contribution of extracellular matrix organization, Cx43 localization, and non-cardiomyocyte interactions.^83^ A related study found reduced contractility in similar multicellular constructs.^56^^,^^83^ Parallel work using patient-derived epicardial cells with the *PKP2*^WT/c.2013delC^ mutation showed spontaneous lipid accumulation exclusively in *PKP2*-mutant epicardial derivatives but not in matched cardiomyocytes, indicating cell-type-specific susceptibilities.^88^

Consistent with this expanding multicellular perspective, single-cell analyses of explanted ACM hearts reveal epicardial activation with elevated TFAP2A, consistent with a shift toward a mesenchymal-like state.^88^ Patient-derived cardiac mesenchymal cells (cMSCs) have been observed to exhibit dysregulated Ca^2+^ signaling and CaMKII activation, which drive fibro-fatty differentiation, an effect partly reversible with flecainide treatment.^90^ Additionally, cMSCs have been shown to undergo extensive epigenetic and metabolic reprogramming, including mitochondrial dysfunction, reactive oxygen species (ROS) accumulation, and activation of epithelial-mesenchymal transition (EMT) and adipogenic pathways.^91^ These findings first implicated cMSCs as active contributors to fibro-adipose remodeling. Building on this, a recent multi-level analysis demonstrated that *PKP2*^WT/c.2013delC^ hiPSC-derived cardiomyocytes secrete reduced levels of the NOTCH antagonist DLK1, resulting in aberrant NOTCH activation in adjacent primary RV-derived cMSCs and inducing adipogenic and fibrotic differentiation.^73^ Under normal physiological conditions, cMSCs help maintain cardiac structural homeostasis and modulate repair, but in *PKP2*-mutant settings, they become active contributors to fibro-adipose remodeling rather than passive responders to cardiomyocyte dysfunction.^92^

Taken together, these studies demonstrate that multicellular hiPSC-based systems can recapitulate hallmark features of ACM, yet phenotype expression is highly context dependent and strongly influenced by cell-type origin and model architecture. Despite major advances, it remains unresolved how fibroblasts, endothelial cells, epicardial derivatives, and cMSCs coordinate with *PKP2*-deficient cardiomyocytes to drive fibro-fatty replacement, conduction abnormalities, and arrhythmia susceptibility. Resolving these interactions will require dynamic multicellular models capable of tracking reciprocal signaling and biomechanical coupling over time.

### Gene therapy applied to hiPSC-derived models

Recent advances in gene therapy have offered promising strategies to restore PP2 levels and rescue the disease phenotype in patient-derived hiPSC models. Since *PKP2* haploinsufficiency directly impairs desmosomal integrity and cardiomyocyte contractility, gene replacement strategies aim to address this core mechanism of the disease.^11^^,^^93^ Three hiPSC models have tried to utilize AAV-based gene therapy thus far.^61^^,^^64^^,^^81^ In a siRNA-mediated *PKP2* knockdown model, AAV-based *PKP2* gene delivery restored desmosomal architecture and contractile function in monolayers within 8 days.^64^ In another monolayer model (*PKP2*^WT/c.1228>NHEJ^/*PKP2*^c.1228>NHEJ/c.1228>NHEJ^), AAV-mediated gene replacement of *PKP2* successfully restored desmosomal proteins and alleviated contractile dysfunction.^81^ Complementing this, AAV6-based gene therapy was applied to *PKP2*^WT/c.2013delC^-engineered cardiac tissues, which increased the expression and localization of key intercalated disc proteins, plakoglobin and desmoplakin, and significantly improved their contractile performance. These results indicate that *PKP2* restoration through AAV therapy may be promising for ACM treatment.^61^

Despite these advances, several key challenges remain. These include improving vector tropism and delivery efficiency, achieving durable and regulated transgene expression, and minimizing immune responses and off-target effects. Moreover, existing hiPSC-based gene therapy models primarily focus on cardiomyocytes, often lacking patient specific non-myocyte populations such as fibroblasts and endothelial cells, much like their murine AAV study counterparts. This limits the ability to assess how gene therapy might influence the broader multicellular cardiac microenvironment involved in arrhythmogenic remodeling.^94^^,^^95^ Finally, despite progress in hiPSC-based models, fundamental aspects of *PKP2*-induced ACM remain unresolved. In particular, the origins of hallmark phenotypes such as fibro-fatty replacement and their contribution to arrhythmia susceptibility require further investigation.^84^^,^^87^^,^^88^

## Discussion

Recent advancements in disease modeling have substantially enhanced our understanding of *PKP2*-induced ACM. Both animal systems and increasingly sophisticated multicellular hiPSC-based platforms now recapitulate important elements of the disease phenotype, but they also reveal persistent gaps in how PP2 deficiency disrupts the complex cellular network of the human myocardium.

Murine studies have been instrumental in defining how PP2 levels influence disease severity. Whole-body *Pkp2* knockout mice revealed that PP2 is required for developmental, whereas heterozygous mice and knockin models exhibited only mild molecular abnormalities without the hallmark arrhythmias or fibro-fatty remodeling, both disease hallmarks that are observed in patients. In contrast, cardiomyocyte-specific conditional *Pkp2* knockout mice and homozygous knockin models more faithfully recapitulate advanced ACM features, including progressive ventricular arrhythmias, fibrosis, chamber dilation, and SCD. The majority of AAV-mediated gene replacement studies were performed in cardiomyocyte-specific murine models and demonstrated robust rescue of cardiomyocyte function. By comparison, the only non-cardiomyocyte-specific *Pkp2* model displayed a very mild baseline phenotype, limiting assessment of therapeutic efficacy and leaving the contribution of non-myocyte populations largely untested. While this bias limits insight into non-myocyte-driven remodeling, it has also enabled precise demonstration that early restoration of cardiomyocyte *Pkp2* levels can prevent or substantially delay disease progression. The relative infrequency with which mice develop early arrhythmias and fibro-fatty infiltration, compared with human ACM, likely reflects these species- and model-specific constraints. Across murine models, a key unresolved issue remains the temporal hierarchy of disease mechanisms, namely whether electrical instability, mechanical uncoupling, or metabolic remodeling represents the initiating event in *Pkp2*-induced ACM, and how this sequence constrains the window during which cardiomyocyte-targeted interventions remain effective.

hiPSC-derived models complement murine studies by capturing patient-specific disease mechanisms and highlighting the intrinsically multicellular nature of *PKP2*-induced ACM. Early EB-based models, representing “undefined” multicellular systems, recapitulated several disease features, including desmosomal disruption, metabolic remodeling, and adipogenic tendencies. However, they could not assign phenotypes to specific cell types. Subsequent patient specific monocellular models, typically using purified hiPSC-cardiomyocytes, enabled controlled research of cardiomyocyte-autonomous mechanisms but frequently lost key ACM characteristics, particularly those emerging from non-myocyte populations. More recently, defined multicellular hiPSC systems incorporating cardiomyocytes together with fibroblasts, endothelial cells, epicardial derivatives, and cMSCs have provided a framework to dissect how individual cardiac cell types contribute to disease initiation and progression. The addition of 3D microtissues and engineered cardiac constructs further revealed that tissue architecture, mechanical load, and cell-cell interactions strongly modulate phenotype expression.

However, even in these advanced 3D systems, most studies capture static endpoints rather than longitudinal disease evolution, limiting insight into the temporal sequence by which electrical, metabolic, and structural abnormalities emerge and interact. While these studies demonstrate that non-myocyte populations actively drive disease processes rather than merely responding to cardiomyocyte dysfunction, interpretation is complicated by differences between 2D monolayers and 3D systems, where architectural and biomechanical context can yield divergent phenotypes. Taken together, these findings indicate that *PKP2*-induced ACM is dose sensitive, with distinct functional thresholds determining whether disease manifests predominantly as electrical dysfunction, mechanical failure, or fibro-fatty remodeling.

This integrated body of work carries direct implications for molecular therapy. Although AAV-mediated *PKP2* or *Pkp2* delivery in cardiomyocyte-specific murine models effectively restores cardiomyocyte function, translation to human disease remains uncertain because non-myocyte contributions are largely unaddressed. The initiation of first-in-human AAV9-*PKP2* clinical trials (TN-401 [AAV9; NCT06228924] and LX2020 [AAVrh10; NCT06109181]) therefore represents a pivotal milestone. While both vectors demonstrate cardiac tropism, AAV9 shows a strong preference for cardiomyocytes, whereas AAVrh10 exhibits broader cardiac enrichment without strict cell type specificity. Importantly, these trials are being conducted predominantly in adult patients, who are likely to have progressed beyond early disease stages.

Insights from murine gene-therapy studies consistently indicate that therapeutic efficacy is strongly dependent on timing, with presymptomatic or very early intervention producing substantially greater and more durable benefit than treatment initiated after structural and electrical remodeling has progressed. These findings align with emerging hiPSC-based evidence that early disease stages may be predominantly cardiomyocyte driven, whereas later phases involve active contributions from fibroblasts, epicardial derivatives, and cardiac mesenchymal cells that promote fibro-fatty replacement and conduction instability. In this context, cardiomyocyte-directed gene therapy may be most effective before irreversible multicellular remodeling is established.

A key translational challenge is that patients enrolled in current clinical trials are typically middle-aged and symptomatic, likely corresponding to more advanced disease stages in which non-myocyte processes are already engaged. Integrating longitudinal clinical outcomes with stage-resolved murine models and mechanistically informative hiPSC systems will therefore be essential to define the therapeutic window in humans and to determine whether early cardiomyocyte-restricted *PKP2* restoration is sufficient, or whether broader strategies that address non-myocyte involvement will be required to fully suppress fibro-fatty remodeling and arrhythmia susceptibility. Given the genetic homogeneity of *PKP2*-mediated ACM and the possibility of identifying at-risk carriers long before symptom onset, these findings also raise important ethical considerations regarding early intervention, in which informed patient participation and shared decision-making will be central to balancing potential benefit against long-term risk.

## Acknowledgments

The authors have no acknowledgments to declare.

## Author contributions

L.G. was responsible for conceptualization, data curation, investigation, visualization, and writing the original draft. Y.R.C. contributed to the conceptualization, investigation, and the writing and editing of the manuscript. M.C.S.C.V. oversaw project design and supervision and also contributed to writing and editing. All authors have read and approved the final version of the manuscript.

## Declaration of interests

The authors declare no competing interests.

## Declaration of generative AI and AI-assisted technologies in the writing process

During the preparation of this work, the authors used DeepL in order to improve the scientific tone of the manuscript, and ChatGPT was used to minimize the word count. After using this tool/service, the authors reviewed and edited the content as needed and take full responsibility for the content of the published article.

## Supplemental information

Table S1. Summary of animal and hiPSC models studying PKP2 variants and their cardiac phenotypes, including structural, functional, and electrophysiological alterations
